# Assessing the calorific significance of episodes of human cannibalism in the Palaeolithic

**DOI:** 10.1038/srep44707

**Published:** 2017-04-06

**Authors:** James Cole

**Affiliations:** 1 School of Environment and Technology, University of Brighton, Cockcroft Building, Lewes Road Brighton, BN2 4GJ, UK

## Abstract

Episodes of Palaeolithic cannibalism have frequently been defined as ‘nutritional’ in nature, but with little empirical evidence to assess their dietary significance. This paper presents a nutritional template that offers a proxy calorie value for the human body. When applied to the Palaeolithic record, the template provides a framework for assessing the dietary value of prehistoric cannibalistic episodes compared to the faunal record. Results show that humans have a comparable nutritional value to those faunal species that match our typical body weight, but significantly lower than a range of fauna often found in association with anthropogenically modified hominin remains. This could suggest that the motivations behind hominin *anthropophagy* may not have been purely nutritionally motivated. It is proposed here that the comparatively low nutritional value of hominin cannibalism episodes support more socially or culturally driven narratives in the interpretation of Palaeolithic cannibalism.

Human cannibalism is a subject that continues to hold a morbid fascination within modern societies. In particular, identifying the motivations for human cannibalism remains a contentious issue. In modern humans, the motivations for cannibalism have been related to any combination of the following: survival; psychotic or criminal; aggressive; spiritual or ritual; gastronomic or dietary; and medicinal^1^,^2^,^3^,^4^. All of these can be further categorised as inter (exo-) and intra-group (endo-) cannibalism, with differing motivational states depending on whether or not the consumed is a member of the consumer’s immediate social network^4^,^5^. Cannibalism is not, however, purely a characteristic of modern humans, and has been practiced by a range of hominin species from at least the early Pleistocene^2^,^6^. The evidence from the archaeological record would suggest that, whilst different hominin species clearly had the capacity for cannibalistic practices, not every hominin population did so, on the basis that not all hominin remains show evidence for anthropogenic modifications. The hominin remains that do exhibit anthropogenic modifications may imply they were cannibalised, although, there are also alternate explanations such as defleshing and excarnation.

Globally, the number of Palaeolithic cannibalism fossil sites remain relatively few^5^, further suporting the notion that the practice of hominin cannibalism may have been an exceptional activity. However, given the sparse nature of the hominin fossil record, the fact that we have evidence for cannibalism at all infers that the behaviour was perhaps more common within prehistoric populations^7^ than the number of archaeological sites suggests. Additional support for the possible widespread nature of prehistoric cannibalism comes from genetic studies of global patterns of transmissible spongiform encephalopathies (TSEs)^8^, which imply that prehistoric TSE polymorphisms were a routine feature of hominin life. Mead *et al*., for example, propose that the repeated exposure of hominins to the effects of TSEs (such as Kuru and Creutzfeldt-Jakob disease) resulting from cannibalistic activities, drove the polymorphism adaptation as a selective advantage within prehistoric populations^8^,^9^. These authors argue that such an adaptation would only be necessary if exposure to the neurodegenerative diseases (through the consumption of infected flesh) was a common feature in prehistoric hominin lifeways.

Our understanding of prehistoric cannibalism has increased exponentially over the last few years thanks to methodological advances and increasing interpretive rigour when examining and recognising anthropogenically modified hominin remains^2^,^10^,^11^,^12^. In the majority of studies, the interpretation is that cannibalism was practiced for nutritional reasons^2^,^5^,^6^,^13^ although there has never been a way to quantify how nutritional these episodes may be. For example, while varied practices of consumption have been identified amongst Neanderthal populations from Moula-Guercy (France)^14^, Cueva del Sidrón (Spain)^15^, Cueva del Boquete de Zafarraya (Spain)^16^, Padrelles (France)^17^,^18^, and Troisième caverne of Goyet (Belgium)^11^, all are broadly interpreted as nutritional. A small number of studies also invoke ritual motivations to, for example, the Upper Palaeolithic episodes of cannibalism associated with *Homo sapiens* at Gough’s Cave (UK)^9^,^10^,^19^ and, less certainly, at the potential *Homo erectus* site of Caune de l’Argo (France)^5^,^20^. Some sites, such as Krapina (Croatia), Brillenhöhle (Germany) and Monte Cicero (Italy), have served as useful cautionary tales, with initial behavioural interpretations of cannibalism being overturned once additional analyses were carried out on the hominin remains^21^,^22^,^23^,^24^ (although the cases of Krapina and Brillenhöhle remain controversial in that they may well now be cannibalism sites^25^,^26^). Other specimens, such as those from Bodo^27^ and Herto^7^,^28^ (Ethiopia), illustrate how our hominin ancestors anthropogenically modified human remains through defleshing; however, it is not clear whether this defleshing was followed by the actual consumption of flesh.

Instances of prehistoric cannibalism have been distinguished within the archaeological record based on anthropogenic modification of hominin skeletal remains in relation to taphonomic processes. The key signatures of cannibalism^1^,^2^,^11^,^14^,^19^,^29^,^30^,^31^,^32^,^33^,^34^,^35^,^36^,^37^ include: 1. lack of a cranial base (to get to the brain) on otherwise complete or near-complete skeletons; 2. virtual absence of vertebrae (due to crushing or boiling to get at bone marrow and grease); 3. cut- and chop-marks; 4. cutmark arrangement: position, number and placement; 5. long bone breakage (to access the marrow); 6. anvil abrasions; 7. comparable butchering techniques on human remains as in faunal (food) remains; 8. post-processing discard of hominin remains similar to faunal remains; 9. evidence of cooking in the form of burnt bone; 10. peeling: a roughened bone surface with parallel grooves or fibrous texture is produced when fresh bone is fractured and peeled apart; 11. percussion pits: the point of impact where a stone or any solid matter struck the bone cortex and scarred the surface; 12. human tooth marks; and 13. scraping marks.

Using a combination of these signatures, archaeologists have determined whether the cannibalism practiced at prehistoric sites was either ‘nutritional’ or ‘ritual’. For example, if signatures 1–12 are present then nutritional cannibalism may be inferred. If 13 is present on cranial remains whilst the rest of the carcass displays 1–12, ritual cannibalism with a special treatment or focus on the crania may be inferred (such as at Gough’s Cave and Caune de l’Argo). It should also be noted that a lack of cranial base could be related to the production of skull-cups^10^,^38^,^39^,^40^. Therefore, the use of the first signature in determining the motivation behind cannibalism acts within the archaeological record should be applied cautiously alongside a majority of signatures 2–12 to infer a motivation beyond ritual – if only signature 13 and/or 1 are present on a hominin carcass, then defleshing of the carcass for secondary burial or some other pre-depositional treatment of the dead (such as skull-cups), may be suggested. Recent work has further demonstrated that distinctions between cannibalism and the secondary treatment of human bodies can be inferred from the micromorphometric characteristics of cutmarks^12^, representing a significant methodological advance in allowing researchers to interpret the motivations behind the acts of prehistoric cannibalism.

Investigations at Atapuerca^6^,^13^ and Gough’s Cave^9^ have demonstrated how Palaeolithic cannibalism interpretations can be extended beyond the broad labels of ‘nutritional’ or ‘ritual’ based on the series of cannibalistic signatures stated above. Indeed, these labels may be seen as somewhat ambiguous given that all types of cannibalism involve feeding on the tissues of individuals of the same species and are therefore inherently ‘nutritional’, regardless of secondary emotive drivers such as ceremony or ritual^5^. Saladié and Rodríguez-Hidalgo^5^ go further in highlighting the often confusing nature of labelling and interpreting episodes of prehistoric cannibalism, and rightly call for a more holistic approach including the use of taphonomy and demography alongside analyses of associated remains (e.g. stone tools), stratigraphy, DNA, isotopes and chronological series data.

This paper offers a new tool to be used in assessing episodes of cannibalism, by presenting for the first time a full nutritional template for the calorific value of the human body in comparison with the faunal record. The use of such a tool will allow researchers to determine how humans compare at a calorie level with other faunal species, and permit the assessment of whether the majority of prehistoric cannibalism claims were genuinely ‘nutritional’ in nature.

## Results: A nutritional template for the human body

Prior to this study, only one published estimate of the nutritional value of the human body seems to have been made. Garn and Block^41^ claimed that a 50 kg male would yield 30 kg of edible muscle mass, which in turn would yield around 4.5 kg of protein or 18,000 calories. However, no information was supplied by which this estimate could be tested or assessed. The authors further suggested that this would serve one day’s protein requirement for 60 people (averaging 60 kg in weight, working on the protein requirement principles that 1 gram of protein is needed per kilogram of body weight per day)^41^. If this were extended to a ‘person a week’ ration for a group of 60 people, then this would amount to 9 grams (36 calories) of quality protein per day. These calculations led the authors to conclude; “the nutritional value of cannibalism may therefore be viewed as questionable, unless a group is in a position to consume its own number in a year”^41^^: 106^.

To construct the human nutritional template in this study, the total average weights and calorie values (fat and protein) for each body part were combined from published chemical composition analyses of four male individuals^42^,^43^,^44^. The published materials used here are the only sources that shared the same original data format, in displaying the full body compositional data as percentages for body weight, fat and protein content. This in turn facilitated a clear comparison of data across the individual specimens. The results are summarised in Table 1, with full methods, calculations and detailed data tables given in Supplementary Information 1 (S1).

Garn and Block’s^41^ original estimations of the calorie value of protein within edible skeletal muscle mass (18,000 calories per 30 kg muscle mass) are not dissimilar to the results obtained from the nutritional template presented in this study (19,951 calories per 24.897 kg muscle mass – S1) although they do seem to have underestimated the overall potential calorie values of skeletal muscle mass. In addition, Garn and Block concentrated solely on skeletal muscle tissue, which is not the only edible component of the human body. From ethnographic and archaeological studies, other body parts are known to be eaten during episodes of cannibalism, including the lungs, liver, brain, heart, nervous tissue, bone marrow, genitalia and skin^1^,^2^,^12^,^14^,^19^,^29^,^30^,^45^. Table 1 therefore shows the full nutritional value of the human body (protein + fat) and highlights the nutritional value of those parts of the body that are most commonly consumed according to ethnographic and archaeological accounts (marked*).

There are some caveats to consider with the nutritional template presented in Table 1. First, the nutritional template represents only the potential nutritional value of an adult human male. Ideally, nutritional templates for females and a range of ages would be constructed, to represent the full nutritional potential of hominin social groups (see discussion). However, data for females and sub-adults are not available within the published literature, and the collection of primary data of this nature was outside the ethical (and legal) scope of this study. Given the proxy nature of the nutritional template, one solution to the age distribution problem is to calculate the weight percent ratio of male infant, child, juvenile, and adolescent to adult, and downscale the proxy calorie value accordingly (Table 2). Male weights were used to fit the parameters of the human nutritional template and taken from the United Kingdom *Royal College of Paediatrics and Child Health* and *World Health Organisation* growth projection charts^46^,^47^. It should be kept in mind that as growth rates are not linear, the values represent a simplified reflection of reality in regards to calorie values. However, the average values presented within the broad age categories in Table 2 (infant, child, juvenile, adolescent and adult) match the age categories used in the archaeological sites under investigation (Table 3) and are therefore useful as a heuristic device when calculating the overall calorie values for episodes of Palaeolithic cannibalism.

A further consideration is that the nutritional values obtained only pertain to modern humans. It is unknown whether the data would change substantially for non-*Homo sapiens* species. In the case of Neanderthals, for example, it is probable that the values for skeletal muscle and related organs would increase given their greater muscle mass^48^. The estimates given in this study should therefore be taken as minimum values for non-*Homo sapiens* hominin species. A third caveat is the use of average values from a small base sample when calculating human calorie values. Due to the variable nature of each human individual this cannot be avoided without a substantially larger dataset (which was unavailable at the time of writing). Finally, the values in Tables 1 and 2 are for raw meat only. There has been much recent interest in how cooking can increase the calorie value retrieved from meat^49^,^50^,^51^. However, given the nature of this study, it was not possible to conduct analyses on cooked human flesh.

## Discussion: Calorific values for episodes of Palaeolithic cannibalism

Having established baseline calorific values for the human body it is now possible to apply those values to a sample of Palaeolithic cannibalism episodes (Table 3). The sites chosen were those highlighted in a recent review on prehistoric cannibalism^5^ that did not have any ambiguity surrounding the interpretation of cannibalism as a behavioural act. Later Prehistoric sites were not included as the focus of this research falls within the Palaeolithic and understanding the motivations of our hominin ancestors for such acts. We know that *Homo sapiens* motivations for cannibalism are frequently context specific, including survival, warfare and symbolic cannibalism as discussed above^5^. Attempting to understand the possible range of motivations for cannibalism in other hominin species therefore forms a focal point of interest here. When estimating the calorific values of the selected cannibalism episodes, three values were assigned per Palaeolithic site (Table 4): (i) A total full body calorie value (using the Total value from Table 1), which can be seen as a maximum value for the episode, (ii) an intermediate value using only the body parts known to be consumed through the ethnographic and archaeological records (*), and (iii) a minimum value where only the skeletal muscle calorie values were applied.

Given that the selected Palaeolithic episodes of cannibalism involved the consumption of individuals across the age spectrum (Table 3), Table 4 has used the age-corrected values (from Table 2) and therefore offers a more realistic calorific value that is used throughout the rest of this study. It should be noted that although the sites in Tables 3 and 4 exhibit anthropogenic modifications on more than 20% of the hominin remains^5^ (with some, such as Gough’s Cave, at over 65% modification^9^), this level of published detail was not available for all sites within this study. To facilitate cross-site comparisons in regards to calories, each site is taken as 100% modification and therefore represents a maximum potential calorie value. In reality, the prehistoric episodes may well have produced less calories based on the degree of consumption and modification of the hominin skeletons.

From Table 4 we can see that there are a range of calorie values per site that correspond directly to the number of individuals being consumed. To assess the nutritional viability of the cannibalism episodes in their broader archaeological context, a comparison is needed with the nutritional value of other faunal species from sites where cannibalism is known to have occurred (Table 3). Table 5 shows the nutritional value of a human body based on skeletal muscle compared to the nutritional value for a number of anthropogenically modified fauna found in close association with hominin remains at the Palaeolithic sites.

Previous studies^52^,^53^ have tended to focus on calorie values for the flesh of the Pleistocene fauna. However, as with the hominin remains, faunal remains are often exploited for additional resources (e.g. bone marrow). Skeletal muscle was used for the nutritional comparison due to a lack of data to facilitate a full body break down of nutritional values across all faunal species. Despite this limitation, the skeletal muscle values serve as a reasonable proxy to assess the calorie values of hominins and other faunal remains. While all the non-carnivorous species from Table 3 are represented in Table 5, there were limited data available (apart from bear) to represent the carnivore remains. Fish and birds are included to represent a scale of low calorie faunal remains that are frequently exploited by humans, even if not directly represented within the assemblages of the sites under study. As with the hominin sites above, the calorie values presented are based on the assumption that 100% of the flesh was consumed to facilitate a direct comparison between faunal and human species. Table 5 shows that when compared to most other fauna, human skeletal muscle has a nutritional value broadly in line with those that match our size and weight, but produce significantly fewer calories than most of the larger fauna such as mammoth, woolly rhino or deer species known to have been regularly consumed by past hominins.

When examining examples of prehistoric cannibalism through the archaeological record, it is difficult to ascertain whether the number of anthropogenically modified individuals represent single or multiple episodes of cannibalism. In this discussion, all episodes are treated as a single episode of cannibalism in line with many of the original site interpretations.

In order to enhance our understanding of the episodes of cannibalism beyond calorie counts, Table 6 shows the number of days a group of twenty-five modern males, Neanderthal males and Pleistocene Anatomically Modern Human males could survive from each Palaeolithic cannibalism episode compared against the faunal record. Males were used to fit the parameters of the nutritional template presented within this study and twenty-five is recognised as being the most desirable group size for mobile foraging populations in terms of reproductive viability and general adaptive significance to hunting and gathering societies^54^,^55^,^56^. In addition, twenty-five has successfully been applied previously to Palaeolithic hunting and gathering groups^55^,^56^,^57^. Average calorie values of 2,400 calories for an adult modern human male^41^; 4,070 calories for an adult Neanderthal male^58^; and 3,788.5 calories for a Pleistocene adult Anatomically Modern Human male^58^ were used to represent the amount of daily energy expenditure. The results in Table 6 should be seen as a heuristic device to aid the nutritional comparison between cannibalism episodes and individual faunal remains.

When Tables 5 and 6 are compared it can be seen that whole cannibalistic episodes hold the same calorific value or less than many individual large faunal species (for example: mammoth, rhinoceros, auroch, bison, cow, bear, horse, giant deer, red deer, musk-ox, deer, boar or reindeer). Therefore, it would seem that the large faunal record offers an overall better calorific return per individual than hominins in terms of energy return. Of course, past hominins also exploited the small faunal record (for example, birds, fish, hare, roe deer and saiga) as a part of their diet and all of which return a lower calorie rate than a hominin. However, the mental and physical effort to hunt a hominin would presumably be much greater than that required for small game given the hominins ability to fight, run and think their way out of the hunt and pursuit in a way that a saiga (for example) simply could not. This then leads to the question of why did hominins engage in the practice of cannibalism if the nutritional return (at an individual and group level) would appear to be significantly less than many individual faunal species that were regularly consumed by these Palaeolithic communities.

Recent studies of Palaeolithic cannibalism^6^,^9^,^11^,^12^,^13^,^14^,^53^ have done much to illustrate that the motivations and social contexts behind episodes of cannibalism go beyond the simplistic ‘nutritional’ or ‘ritual’ label. For *Homo sapiens*, the motivations for cannibalism are clearly wide-ranging, including nutritional cannibalism with ritual practices surrounding the special treatment of skulls^9^ and inter-group rivalries placed under stress during harsh climatic conditions^53^,^59^. In regards to Neanderthals there is an increasing body of evidence that suggests they may well have been as socially complex and varied on an intra- and inter-group level as modern humans in the treatment of their dead^60^,^61^,^62^ and within the symbolic realm^63^,^64^,^65^. The site of Caune de l’Argo highlights the intriguing possible nature of ritual cannibalism for *Homo erectus* where the post-cranial remains have been processed in a different fashion to the cranial remains, perhaps facilitating an interpretation not dissimilar to the Gough’s Cave assemblage; although more work on this site is needed to confirm this.

This study demonstrates that on a nutritional level, hominins fall where expected, in terms of calorie content (Table 5), when compared to fauna of a similar body weight. However, when compared to large fauna often found in association with anthropogenically modified hominin remains (Table 3), the calorie returns of individuals and groups of hominins are significantly less than individual large fauna commonly exploited by hominins in the past. So, why cannibalise a member of your own species? Hominins may have been seen (rather functionally) as another source of food (“meat for meat’s sake”) and were cannibalised on an opportunistic basis (such as when a member of the group passed away) possibly as an easy alternative to going out and hunting. Or, perhaps hominins were actively hunted. Active hunting raises the interesting question of whether the relatively low calorific return for hominins would justify the energy expenditure in hunting an individual or group if the motivation was driven purely by balancing energy quotients. It is suggested here that this would not be the case, when a single large fauna individual returns many more calories without the difficulties of hunting groups of hominins that were as intelligent and resourceful as the hunters (in their ability to fight back and evade pursuit). Rather, given the apparent scarcity of cannibalistic behaviour in the archaeological record within individual hominin populations, coupled with a picture of increasing social complexity from hominins during the early Pleistocene onwards, it is more likely that the motivations for cannibalistic episodes lay within complex cultural systems involving both intra-and inter-group dynamics and competition^6^,^13^,^20^. Certainly, this conclusion would support interpretations from Gran Dolina relating to *Homo antecessor*^6^,^13^. The intriguing possibility of *Homo erectus* ritual cannibalism from l’Argo^20^ could further suggest that even the oldest episodes of cannibalism were social acts that had some cultural meaning for the consumers beyond an easy meal.

## Conclusion

Undoubtedly, each episode of Palaeolithic cannibalism would have had its own specific cultural context and reason for consumption. In some instances, this may represent a more practical or opportunistic approach to food procurement, for example, the consumption of individuals who die of natural causes within the social group. Such an interpretation cannot be entirely dismissed given that the nutritional value of the human body is not particularly high, and hominins regularly exploited faunal remains that were lower in calories with no cultural influence. However, the similarity of demographics across Palaeolithic cannibalism episodes (adults to infants) may indicate that the motivations followed the inter- and intra-group dynamics involving resource and territory defence proposed at Gran Dolina TD6^13^. If this is the case, it would suggest that pre-*Homo sapiens* Pleistocene hominin social structures and interactions within and between groups may have been far more complex than currently estimated. Recent palaeo-genetic studies^66^,^67^,^68^ have already hinted at a more explicit and active degree of social interaction between hominin species than was previously thought possible. In addition, the recognised complexity within Neanderthal societies with distinct cultural and symbolic traditions^69^,^70^,^71^ illustrates a hominin that is more behaviourally similar to our own species. We know that modern humans have a range of complex motivations for cannibalism that extend from ritual, aggressive, and survival to dietary reasons. Why then would a hominin species such as the Neanderthals, who seem to have had varying attitudes to the burial and treatment of their dead^22^,^60^,^61^,^62^,^72^, not have an equally complex attitude towards cannibalism? As such, social motivations behind acts of Palaeolithic cannibalism should not be readily discounted when examined within the broader behavioural context of the hominins under study.

The use of the human nutritional template presented here highlights that humans (and by inference hominins) fall within the expected range of calories for an animal of our average body weight. We are, however, significantly lower in calorie value when compared to single large fauna (such as mammoth, bison, cattle and horse) that have a much greater calorific return per individual than many of the groups of cannibalised human remains. This return must therefore question the viability of hunting and consuming hominins for strictly nutritional reasons. It is recommended that the data and methods presented here form part of a holistic approach to the definition of episodes of prehistoric cannibalism, with a stricter use of terminology when describing episodes of prehistoric cannibalism beyond the ambiguous and leading terms ‘nutritional’ or ‘symbolic’.

## Method

The human nutritional template was constructed using previously published data relating to the chemical composition of the human body for four adult male human individuals^42^,^43^,^44^ and average limb muscle weights^73^,^74^. The chemical composition data sources shared the same original data format of displaying the chemical composition of body components as percentages of body weight, fat and protein. In order to obtain the calorie values for each body component, the percentage values had to be converted back to real weights (in grams). The calorie conversion was attained through the ratio 4:4:9, where 1 gram of protein equals 4 calories, 1 gram of carbohydrate equals 4 calories and 1 gram of fat equals 9 calories^75^,^76^. See Supplementary Information 1 for full calculations, data tables and figures.

## Additional Information

**How to cite this article:** Cole, J. Assessing the calorific significance of episodes for human cannibalism in the Palaeolithic. *Sci. Rep.* **7**, 44707; doi: 10.1038/srep44707 (2017).

**Publisher's note:** Springer Nature remains neutral with regard to jurisdictional claims in published maps and institutional affiliations.

## Supplementary Material

Supplementary Information

## Figures and Tables

**Table 1:** Average weight and calorific values for parts of the human body.

Body Component	Average Weight (kg)	Nutritional Value in Calories (Fat + Protein)
Skeletal Muscle [total]:	[24.90]	[32375.50]
*Torso and Head	4.17	5418.67
*Upper arms	5.73	7451.16
*Forearms	1.28	1664.48
*Thighs	10.27	13354.88
*Calves	3.45	4486.30
*Brain, Spinal Cord, Nerve Trunks	1.69	2706.00
*Lungs	2.06	1596.50
*Heart	0.44	650.75
*Kidneys	0.35	376.00
*Liver	1.88	2569.50
*Adipose Tissue	8.72	49938.50
*Skin	4.91	10278.00
*Skeleton	10.31	25331.50
Teeth	0.04	36.00
Nerve Tissue	1.53	2001.00
Alimentary tract	1.23	1263.25
Spleen	0.15	128.33
Pancreas	0.09	160.50
Remaining Tissue: Liquid	1.03	469.50
Solid	6.66	13890.50
Total	65.99	143771.33
Total*	55.26	125822.25

*Parts of the human body reasonably assumed to have been consumed on a regular interval based on ethnographic^4^,^45^ and archaeological sources (see Table 3).

**Table 2:** Estimated total calorie values for male adults, adolescents, juveniles and infants.

	Male adult(18 + years)	Male adolescent(11–18 years)	Male juvenile(7–11 years)	Male child(4–7 years)	Male infant(0–4 years)
Average Weight (kg)	65.99^a^	50.31^b^	28.74^b^	19.85^b^	13.52^c^
% of Adult weight	100.00	76.23	43.55	30.08	20.48
Calorie value total body	143771.33^d^	109596.88	62612.41	32966.74	12823.02
Calorie value * estimates from Table 1	125822.25^d^	95914.30	41770.68	12564.62	2573.23
Calorie value skeletal muscle	32375.50^d^	24679.84	10748.07	3233.02	662.12

^a^Average value used in this study from sample (S1) – 18+ weight from ^b^is 67 kg and thought to be comparable with the average weight from this study (65.99 kg), therefore no corrections were necessary. ^b^Values obtained from^47^ based on average weights per age (4–18) on the 50^th^ centile line. ^c^ Values obtained from^46^ based on average weights per age (0–4) on the 50^th^ centile line. ^d^Calorie values from Table 1.

**Table 3:** Documented sites of Palaeolithic cannibalism.

Site	Date (BP)	Hominin Type	Cannibalism interpretation	Hominin MNI	Age Range of Individuals (following^5^: Table 1)	Associated faunal remains (excluding indeterminate remains)
Gran Dolina (TD6 - Aurora Stratum)^5^,^6^,^13^,^77^,^78^	c. 936,000 BP	*H. antecessor*	Nutritional cannibalism	11	2 adults, 3 adolescents, 6 children	*Cervus, Sus, Equus, Bison, Megaloceros, Dama, Capreolus, Eucladoceros, Stephanorhinus, Mammuthus, Canis, Vulpes, Ursus, Crocuta, Lynx*
Caune de l’Argo^5^,^20^	c. 680,000 BP	*H. erectu*s(?)	Ritual cannibalism (?)	30	18 adults, 12 infants	*Equus, Rangifer, Ovis, Bison, Ovibos, Cervus, Coelodonta*
Moula-Guercy^5^,^14^	100,000 - 120,000 BP	*H. neanderthalensis*	Nutritional or starvation cannibalism	6	2 adults, 2 adolescents, 2 infants	*Cervus, Capra, Artiodactyla* (undefined), *Perissodactyla* (undefined), *Carnivora* (undefined)
El Sidrón^5^,^15^,^79^,^80^,^81^,^82^	48,400 ± 3200 BP	*H. neanderthalensis*	Nutritional or starvation cannibalism	13	7 adults, 3 adolescents, 2 juveniles, 1 infant	Faunal evidence scarce - comparison not possible
Padrelles^5^,^17^,^18^,^83^	c. 45,000 BP	*H. neanderthalensis*	Nutritional cannibalism	5	3 adults, 1 adolescent, 1 infant	*Panthera, Canis, Vulpes/Alopex, Crocuta, Sus, Bovinae, Rangifer, Cervus, Equus, Lepus*
Cueva del Boquete de Zafarraya^5^,^16^,^84^	c. 42,000 BP	*H. neanderthalensis*	Nutritional cannibalism	9	7 adults, 2 infants	*Capra, Bos, Cervus, Sus, Equus, Rupicapra, Panthera, Lynx, Felis, Crocuta, Cuon, Vulpes, Ursus*
Troisième caverne of Goyet^11^,^85^	40,500 - 45,500 cal BP	*H. neanderthalensis*	Nutritional cannibalism	5	4 adults/adolescents, 1 infant	*Equus, Rangifer, Cervus, Capreolus, Megaloceros, Bos, Capra, Sus, Lepus, Mammuthus, Ursus, Vulpes, Crocuta, Meles*
Maszycka Cave^53^	14,280 - 15,800 BP	*H. sapiens*	Warfare cannibalism	16	5 adults, 3 juveniles, 8 infants	*Equus, Cervus, Saiga, Bos, Ursa, Lepus, Sus, Rhinoceros (Ceratotherium*?)
Gough’s Cave^5^,^9^,^10^,^19^	14,700 cal BP	*H. sapiens*	Nutritional cannibalism with ritual treatment of the skulls	5	2 adults, 2 adolescents, 1 infant	*Equus, Cervus, Bos, Sus, Lepus*

MNI = minimum number of individuals.

**Table 4:** Calorific value of each episode of Palaeolithic cannibalism.

Site	^+^Nutritional value based on whole body (calories)	^+^Nutritional value based on * data in Table 1 (calories)	^+^Nutritional value based on skeletal muscle (calories)
Gran Dolina (TD6 - Aurora Stratum)	465549	338237	87032
Caune de l’Argo	2741760	2295679	590704
Moula-Guercy	532382	448620	115435
El Sidrón	898152	751324	193324
Padrelles	553734	475954	122468
Cueva del Boquete de Zafarraya	1032045	885902	227953
Troisième caverne of Goyet	587908	505862	130164
Maszycka Cave	1009278	775009	199419
Gough’s Cave	519559	446046	114773

^+^Calorie values (Table 1) incorporating the age corrected estimates from Table 2 as per Table 3. All calorie values have been rounded to the nearest whole value.

**Table 5:** Average total weight values, average muscle weight and average calorie values for muscle weight of faunal and human species based on available data.

Hominin/Fauna	Average Total Weight (kg)	Average Muscle Weight (kg)*	Calorie/kg (muscle)	Calorie Value (muscle)
*Hominin (H. sapiens*)	65.99	24.9	1300	32376
Mammoth (Mammuthus)	3000	1800	2000	3600000
Woolly Rhinoceros (*Coelodonta/Stephanorhinus/Rhinoceros*)	1200	720	1750	1260000
Aurochs (*Bos*)	800	480	2040	979200
Steppe Bison (*Bison*)	500	300	2040	612000
Cattle sp. (*Bos*)	300	180	2040	367200
Bear (*Ursus*)	250	150	4000	600000
Horse sp. (*Equus*)	290	174	1150	200100
Giant deer (*Megaloceros/Eucladoceros*)	220	132	1240	163680
Red deer (*Cervus*)	220	132	1240	163680
Musk-ox (*Ovibos*)	180	108	1300	140400
Deer sp. (*Cervus/Dama*)	160	96	1240	119040
Boar (*Sus*)	135	81	4000	324000
Reindeer (*Rangifer*)	100	60	1000	60000
Ibex (*Capra*)	70	42	1090	45780
Rupicapra (*Rupicapra*)	60	36	1045	37620
Saiga (*Saiga*)	45	31.5	1000	31500
Roe deer (*Capreolus*)	22	13.2	1000	13200
Beaver (*Castor*)	20	12	4000	48000
Hare sp. (*Lepus*)	6	3.6	1070	3852
Steppe marmot (*Marmota*)	1	0.6	3000	1800
Fish	1.25	1	1300	1300
Birds	1.25	1	2500	2500

Faunal muscle and calorie values from^52^: 294, Table 5. 17 and references therein^53^^: 174, Table 8^, human muscle values from this study. All calorie values have been rounded to the nearest whole number. Giant deer (*Megaloceros/Eucladoceros*) estimated as red deer, and Deer sp. estimated as median between red deer and reindeer as per^52^. Ibex calorie per kg estimated the same as goat^86^; Rupicapra estimated as median between Ibex and Saiga; Roe deer calorie per kg estimated the same as Saiga. *Estimated as 60% of average total weight for mammals and 80% of average total weight for fish and birds after^52^,^87^. *Carnivora* species were excluded due to insufficient data on potential calorie sources per kg weight.

**Table 6:** Comparison of Palaeolithic cannibalism episodes versus faunal remains in regards to calorie content and potential number of days of food provision.

		Skeletal muscle calorie value	Number of days a group of 25 modern human adult males could survive (2,400 calories per person per day)	Number of days a group of 25 adult Neanderthal males could survive (4,070 calories per person per day*)	Number of days a group of Pleistocene anatomically modern humans could survive (3,788.5 calories per person per day*)
Site	Gran Dolina (TD6 - Aurora Stratum)	87032	1.45	0.86	0.92
	Caune de l’Argo	590704	9.85	5.81	6.24
	Moula-Guercy	115435	1.92	1.13	1.22
	El Sidrón	193324	3.22	1.90	2.04
	Padrelles	122468	2.04	1.20	1.29
	Cueva del Boquete de Zafarraya	227953	3.80	2.24	2.41
	Troisième caverne of Goyet	130164	2.17	1.28	1.37
	Maszycka Cave	199419	3.32	1.96	2.11
	Gough’s Cave	114773	1.91	1.13	1.21
Fauna/Hominin	Hominin (*Homo sapiens*)	32376	0.54	0.32	0.34
	Mammoth (*Mammuthus*)	3600000	60.00	35.38	38.01
	Woolly Rhinoceros (*Coelodonta/Stephanorhinus/Rhinoceros*)	1260000	21.00	12.38	13.30
	Aurochs (*Bos*)	979200	16.32	9.62	10.34
	Steppe Bison (*Bison*)	612000	10.20	6.01	6.46
	Cattle sp. (*Bos*)	367200	6.12	3.61	3.88
	Bear (*Ursus*)	600000	10.00	5.90	6.33
	Horse sp. (*Equus*)	200100	3.34	1.97	2.11
	Giant deer (*Megaloceros/Eucladoceros*)	163680	2.73	1.61	1.73
	Red deer (*Cervus*)	163680	2.73	1.61	1.73
	Musk-ox (*Ovibos*)	140400	2.34	1.38	1.48
	Deer sp. (*Cervus/Dama*)	119040	1.98	1.17	1.26
	Boar (*Sus*)	324000	5.40	3.18	3.42
	Reindeer (*Rangifer*)	60000	1.00	0.59	0.63
	Ibex (*Capra*)	45,780	0.76	0.45	0.48
	Rupicapra (*Rupicapra*)	37620	0.63	0.37	0.40
	Saiga (*Saiga*)	31500	0.53	0.31	0.33
	Roe deer (*Capreolus*)	13200	0.22	0.13	0.14
	Beaver (*Castor*)	48000	0.80	0.47	0.51
	Hare sp. (*Lepus*)	3852	0.06	0.04	0.04
	Steppe marmot (*Marmota*)	1800	0.03	0.02	0.02
	Fish	1300	0.02	0.01	0.01
	Birds	2500	0.04	0.02	0.03

*Calorie values for Neanderthals and Pleistocene Anatomically Modern Humans were calculated as the combined average of the Daily Energy Expenditure (DEE) values for Cold and Temperate climates given in Froehle and Churchill^58^ Table 5.

## References

[b1] Villa P. . Cannibalism in the Neolithic. Science 233, 431–437 (1986). 17794567 10.1126/science.233.4762.431

[b2] Fernandez-Jalvo Y., Diez J. C., Caceres I. & Rosell J. Human cannibalism in the Early Pleistocene of Europe (Gran Dolina, Sierra de Atapuerca, Burgos, Spain). Journal of Human Evolution 37, 591–622 (1999). 10497001 10.1006/jhev.1999.0324

[b3] Goldberg H. Cannibalism in Iberian Narrative: The Dark Side of Gastronomy. Bulletin of Hispanic Studies 74, 107–122 (1997).

[b4] Vilaca A. Relations between Funerary Cannibalism and Warfare Cannibalism: The Question of Predation. Ethnos 65, 84–106 (2000).

[b5] Saladié P. & Rodriguez-Hidalgo A. Archaeological Evidence for Cannibalism in Prehistoric Western Europe: from Homo antecessor to the Bronze Age. Journal of Archaeological Method and Theory, doi: 10.1007/s10816-016-9306-y (2016).

[b6] Saladié P. . Intergroup cannibalism in the European Early Pleistocene: The range expansion and imbalance of power hypotheses. Journal of Human Evolution 63, 682–695 (2012). 22944348 10.1016/j.jhevol.2012.07.004

[b7] Stringer C. B. The Origin of Our Species (Allen Lane, 2011).

[b8] Mead S. . Balancing Selection at the Prion Protein Gene Consistent with Prehistoric Kuru like Epidemics. Science 300, 640–643 (2003). 12690204 10.1126/science.1083320

[b9] Bello S. M., Saladié P., Caceres I., Rodríguez-Hidalgo A. & Parfitt S. A. Upper Palaeolithic ritualistic cannibalism at Gough’s Cave (Somerset, UK): The human remains from head to toe. Journal of Human Evolution 82, 170–189 (2015). 25887278 10.1016/j.jhevol.2015.02.016

[b10] Bello S. M., Parfitt S. A. & Stringer C. B. Earliest directly-dated human skull-cups. PLoS One 6, e17026 (2011). 21359211 10.1371/journal.pone.0017026 PMC3040189

[b11] Rougier H. . Neanderthal cannibalism and Neanderthal bones used as tools in Northern Europe. Nature: Scientific Reports 6, 29005, doi: 10.1038/srep29005 (2016). PMC4933918 27381450

[b12] Bello S. M., Wallduck R., Dimitrijevic V., Zivaljevic I. & Stringer C. B. Cannibalism versus funerary defleshing and disarticulation after a period of decay: comparisons of bone modifications from four prehistoric sites. American Journal of Physical Anthropology, doi: 10.1002/ajpa.23079 (2016). 27561127

[b13] Carbonell E. . Cultural Cannibalism as a Paleoeconomic System in the European Lower Pleistocene: The Case of Level TD6 of Gran Dolina (Sierra de Atapuerca, Burgos, Spain). Current Anthropology 51, 539–549 (2010).

[b14] Defleur A., White T., Valensi P., Slimak L. & Crégut-Bonnoure E. Neanderthal Cannibalism at Moula-Guercy, Ardèche, France. Science 286, 128–131 (1999). 10506562 10.1126/science.286.5437.128

[b15] Rosas A. . Paleobiology and comparative morphology of a late Neandertal sample from El Sidron, Asturias, Spain. Proceedings of the National Academy of Sciences 103, 19266–19271 (2006). 10.1073/pnas.0609662104 PMC1748215 17164326

[b16] Barroso C. & de Lumley H. La Grotte du Boquete de Zafarraya (Junta de Andalucia, 2006).

[b17] Maureille B. . Le gisement mousterien des Pradelles (Marillac-le-Franc, Charente): passe, present, futur. XXVI Congres Prehistorique de France: Congres du Centenaire, Societe Prehistorique Francaise 249–261 (2007).

[b18] Mussini C. *Les restes humains moustériens des Pradelles (Marillac-le-Franc, Charente, France): étude morphométrique et réflexions sur un aspect comportemental des Néandertaliens* Université Bordeaux 1 (2011).

[b19] Andrews P. & Fernandez-Jalvo Y. Cannibalism in Britain: Taphonomy of the Creswellian (Pleistocene) faunal and human remains from Gough’s Cave. Bulletin of the Natural History Museum: Geology Series 58, 59–81 (2003).

[b20] de Lumley M.-A. L’homme de Tautavel. Un Homo erectus européen évolué. Homo erectus tautavelensis. L’Anthropologie 119, 303–348 (2015).

[b21] Trinkaus E. Cannibalism and burial at Krapina. Journal of Human Evolution 14, 203–216 (1985).

[b22] Russell M. D. Mortuary practices at the Krapina Neanderthal site. American Journal of Physical Anthropology 72, 381–397 (1987). 3107399 10.1002/ajpa.1330720311

[b23] White T. D. & Toth N. The question of ritual cannibalism at Grotta Guattari. Current Anthropology 32, 118–138 (1991).

[b24] Orschiedt J. Secondary burial in the Magdalenian: the Brillenhöhle (Blaubeuren, Southwest Germany). Palaeo 14, 241–256 (2002).

[b25] White T. D. & Toth N. In Breathing Life into Fossils: Taphonomic Studies in Honor of C.K. (Bob) Brain (eds Pickering T. R., Schick K. & Toth N.) 281–296 (Stone Age Press, 2007).

[b26] Sala N. & Conard N. Taphonomic analysis of the hominin remains from Swabian Jura and their implications for the mortuary practices during the Upper Paleolithic. Quaternary Science Reviews 150, 278–300 (2016).

[b27] White T. D. Cutmarks on the Bodo Cranium: A case of Prehistoric Defleshing. American Journal of Physical Anthropology 69, 503–509 (1986). 3087212 10.1002/ajpa.1330690410

[b28] White T. D. . Pleistocene Homo sapiens from Middle Awash, Ethiopia. Nature, 742–747 (2003). 10.1038/nature01669 12802332

[b29] White T. D. Prehistoric Cannibalism at MANCOS 5MTUMR-2346 (Princeton University Press, 1992).

[b30] Turner C. G. II Cannibalism in Chaco Canyon: the charnel pit excavated in 1926 at Small House Ruin by Frank H. H. Roberts. American Journal of Physical Anthropology 91, 421–439 (1993). 8372934 10.1002/ajpa.1330910403

[b31] Hillson S. Cannibalism and Violence. International Journal of Osteoarchaeology 10, 1–3 (2000).

[b32] Taylor T. The Buried Soul: How Humans Invented Death (Fourth Estate, 2002).

[b33] Cole J. Consuming Passions: Reviewing the Evidence for Cannibalism within the Prehistoric Archaeological Record. *Assemblage* http://www.assemblage.group.shef.ac.uk/issue9/cole.html(2006).

[b34] Cole J. *Prehistoric Cannibalism: an act of nutritional necessity or a result of socio-cultural conditions*? MA thesis, University of Southampton (2006).

[b35] Cáceres I., Lozano M. & Saladié P. Evidence for Bronze Age cannibalism in El Mirador Cave (Sierra de Atapuerca, Burgos, Spain). American Journal of Physical Anthropology 133, 899–917 (2007). 17492670 10.1002/ajpa.20610

[b36] Fernandez - Jalvo Y. & Andrews P. When humans chew bones. Journal of Human Evolution 60, 117–123 (2011). 20951407 10.1016/j.jhevol.2010.08.003

[b37] Saladié P., Rodríguez-Hidalgo A., Díez C., Martín-Rodríguez P. & Carbonell E. Range of bone modifications by human chewing. Journal of Archaeological Science 40, 380–397 (2013).

[b38] Massola A. A Victorian skull-cup drinking bowl. Mankind 5, 415–419 (1961).

[b39] Boulestin B. . Mass cannibalism in the Linear Pottery Culture at Herxheim (Palatinate, Germany). Antiquity 83, 968–982 (2009).

[b40] Orschiedt J. & Haidle M. N. The LBK enclosure of Herxheim. Theatre of war or ritual centre? References from osteoarchaeological investigation. Journal of Conflict Archaeology 2, 153–167 (2006).

[b41] Garn S. M. & Block W. D. The limited nutritional value of cannibalism. American Anthropologist 72, 106 (1970).

[b42] Mitchell H. H., Hamilton T. S., Steggerda F. R. & Bean H. W. The Chemical Composition of the Adult Human Body and its bearing on the Biochemistry of Growth. Journal of Biological Chemistry 158, 625–637 (1945).

[b43] Forbes R. M., Cooper R. H. & Mitchell H. H. The Composition of the Adult Human Body as Determined by Chemical Analysis. Journal of Biological Chemistry 203, 359–366 (1953). 13069519

[b44] Forbes R. M., Mitchell H. H. & Cooper R. H. Further Studies on the Gross Composition and Mineral Elements of the Adult Human Body. Journal of Biological Chemistry 223, 969–975 (1956). 13385244

[b45] Walens S. & Wagner R. Pigs, Proteins, and People-Eaters. American Anthropologist 73, 269 (1971).

[b46] RCPCH. Boys UK-WHO Growth Chart 0–4 years. Royal College of Paediatrics and Child Health, World Health Organisation Department of Health (2009).

[b47] RCPCH. Boys UK Growth Chart 2–18 years. Royal College of Paediatrics and Child Health, World Health Organisation Department of Health (2012).

[b48] Churchill S. E. & Rhodes J. A. How strong were the Neanderthals? Leverage and Muscularity at the Shoulder and Elbow in Mousterian Foragers. Periodicum Biologorum 108, 457–470 (2006).

[b49] Wrangham R. Catching Fire: How Cooking Made us Human (Profile Books, 2010).

[b50] Wrangham R. W., Jones J. H., Laden G., Pilbeam D. & Conklin-Brittain N. L. The raw and the stolen: cooking and the ecology of human origins. Current Anthropology 40, 567–594 (1999). 10539941

[b51] Wrangham R. W. In Evolution of the Human Diet: The Known, the Unknown, and the Unknowable (ed. Ungar P. S.) 308–323 (Oxford University Press, 2007).

[b52] Soffer O. The Upper Palaeolithic of the Central Russian Plain (Academic Press, 1985).

[b53] Kozlowski S. K. . Maszycka Cave, a Magdalenian site in Southern Poland. Jahrbuch Römisch-Germanisches Zentralmuseum 40, 115–252 (1995).

[b54] Birdsell J. B. In Man the Hunter (eds Lee R. B. & DeVore I.) 229–240 (Aldine, Chicago, 1968).

[b55] Wobst H. M. Locational relationships in Palaeolithic Society. Journal of Human Evolution 5, 49–58 (1976).

[b56] Wobst H. M. Boundary Conditions for Palaeolithic Social Systems: A Simulation Approach. American Antiquity 39, 147–178 (1974).

[b57] Gamble C. S. The Palaeolithic Societies of Europe (Cambridge University Press, 1999).

[b58] Froehle A. W. & Churchill S. E. Energetic Competition between Neanderthals and Anatomically Modern Humans. Palaeoanthropology, 96–116 (2009).

[b59] Kozlowski S. K., Poltowicz-Bobak M., Bobak D. & Terberger T. New information from Maszycka Cave and the Late Glacial recolonisation of Central Europe. Quaternary International 272–273, 288–296 (2012).

[b60] Rendu W. . Evidence supporting an intentional Neanderthal burial at La Chapelle-aux-Saints. Proceedings of the National Academy of Sciences 111, 81–86 (2014). 10.1073/pnas.1316780110 PMC3890882 24344286

[b61] Sandgathe D. M., Dibble H. L., Goldberg P. & McPherron S. The Roc de Marsal Neanderthal Child: A reassessment of its status as a deliberate burial. Journal of Human Evolution 61, 243–253 (2011). 21664649 10.1016/j.jhevol.2011.04.003

[b62] Pettitt P. B. The Neanderthal dead: exploring mortuary variability in Middle Palaeolithic Eurasia. Before Farming 1, 1–26 (2002).

[b63] Hublin J.-J. . Radiocarbon dates from the Grotte du Renne and Saint-Césaire support a Neandertal origin for the Châtelperronian. Proceedings of the National Academy of Sciences 109, 18743–18748 (2012). 10.1073/pnas.1212924109 PMC3503158 23112183

[b64] Radovčić D., Sršen A. O., Radovčić J. & Frayer D. W. Evidence for Neandertal Jewelry: Modified White-Tailed Eagle Claws at Krapina. PLoS One 10, e0119802, doi: 10.1371/journal.pone.0119802 (2015). 25760648 PMC4356571

[b65] Zilhão J. Personal Ornaments and Symbolism Among the Neanderthals. Developments in Quaternary Science 16, 35–49 (2012).

[b66] Reich D. . Genetic history of an archaic hominin group from Denisova Cave in Siberia. Nature 468, 1053–1060 (2010). 21179161 10.1038/nature09710 PMC4306417

[b67] Meyer M. . A mitochondrial genome sequence of a hominin from Sima de los Huesos. Nature 505, 403–406, doi: 10.1038/nature12788 (2014). 24305051

[b68] Meyer M. . Nuclear DNA sequences from the Middle Pleistocene Sima de los Huesos hominins. Nature 531, 504–507 (2016). 26976447 10.1038/nature17405

[b69] Ruebens K., McPherron S. P. & Hublin J.-J. Regional Behaviour Among Late Neanderthal Groups in Western Europe: A Comparative Assessment of Late Middle Palaeolithic Bifacial Tool Variability. Journal of Human Evolution 65, 341–362 (2013). 23928352 10.1016/j.jhevol.2013.06.009

[b70] Radovčić D., Sršen A. O., Radovčić J. & Frayer D. W. Evidence for Neanderthal Jewelry: Modified White-Tailed Eagle Claws at Krapina. PLOS One, doi: 10.1371/journal.pone.0119802 (2015). PMC4356571 25760648

[b71] Zilhāo J. . Symbolic Use of Marine Shells and Mineral Pigments by Iberian Neandertals. Proceedings of the National Academy of Sciences (PNAS) 107, 1023–1028 (2010). 10.1073/pnas.0914088107 PMC2824307 20080653

[b72] Pettitt P. The Palaeolithic Origins of Human Burial (Routledge, 2010).

[b73] Wang W. . Regional skeletal muscle measurement: evaluation of new dual energy X-ray absorptiometry model. Journal of Applied Physiology 87, 1163–1171 (1999). 10484591 10.1152/jappl.1999.87.3.1163

[b74] Sugawara J. . Age-related reductions in appendicular skeletal muscle mass: association with habitual aerobic exercise status. Clinical Physiology and Functional Imaging 22, 169–172 (2002). 12076341 10.1046/j.1475-097x.2002.00413.x

[b75] Solomon E. P., Schmidt R. R. & Adragna P. J. (Saunders College Publishing, USA, 1990).

[b76] USDA. in *United States Department of Agriculture National Nutrient Database for Standard Reference, Release 18* (2005).

[b77] Bermúdez de Castro J. M. . New immature hominin fossil from European Lower Pleistocene shows the earliest evidence of a modern human dental pattern. Proceedings of the National Academy of Sciences 102, 5674–5678 (2010). 10.1073/pnas.1006772107 PMC2900696 20547843

[b78] Parés J. M. . Reassessing the age of Atapuerca TD-6 (Spain): New paleomagnetic data. Journal of Archaeological Science 40, 4586–4595 (2013).

[b79] Lalueza-Fox C. . Genetic evidence for patrilocal mating behavior among Neandertal groups. Proceedings of the National Academy of Sciences 108, 250–253 (2011). 10.1073/pnas.1011553108 PMC3017130 21173265

[b80] Rosas A. . Les Neanderthaliens d’El Sidron (Asturies, Espagne). Actualisation d’un nouvel echantillon, L’Anthropologie 116, 57–76 (2012).

[b81] Rosas A. . Identification of Neandertal individuals in fragmentary fossil assemblages by means of tooth associations: The case of El Sidrón (Asturias, Spain). Comptes Rendus Palevol 12, 279–291 (2013).

[b82] Wood R. E. . A new date for the Neanderthals from El Sidrón cave (Asturias, Northern Spain). Archaeometry 55, 148–158 (2013).

[b83] Costamagno S. . Homme ou carnivores? Protocole d’etude d’ensembles osseux mixtes: l’exemple du gisement mousterien des Pradelles (Marillac-le-Franc, Charente). Archaeofauna 14, 43–68 (2005).

[b84] Barroso Ruiz C. & Hublin J.-J. In *Gibraltar during the Quaternary* 61–70 (AEQUA Monografias 2) (1994).

[b85] Vallverdú J. . Short Human Occupations in the Middle Palaeolithic level i of the Abric Romaní Rock-Shelter (capellades, Barcelona, Spain). Journal of Human Evolution 48, 157–174 (2005). 15701529 10.1016/j.jhevol.2004.10.004

[b86] USDA. United States Department of Agriculture: Agricultural Research Service, https://ndb.nal.usda.gov/ndb/foods/show/5267?man=&lfacet=&count=&max=50&qlookup=goat&offset=&sort=default&format=Abridged&reportfmt=other&rptfrm=&ndbno=&nutrient1=&nutrient2=&nutrient3=&subset=&totCount=&measureby=&Qv=100&Q9719=1&Q9720=1&Qv=1&Q9719=1&Q9720=1 (2016).

[b87] Wing E. S. & Brown A. B. Paleonutrition: Method and theory in prehistoric foodways (Academic Press, 1979).

