# Supplementary material for: Assessing the calorific significance of episodes of human cannibalism in the Palaeolithic
Source: Sci Rep. 2017 Apr 6;7:44707. doi: 10.1038/srep44707 (PMC5382840; doi:10.1038/srep44707)
Supplement: Supplementary Information [file srep44707-s1.pdf]

Assessing the calorific significance of episodes of human cannibalism in the Palaeolithic

Author:

Dr James Cole, Senior Lecturer in Archaeology, School of Environment and Technology, University of Brighton, Cockcroft Building, Lewes Road, Brighton, BN2 4GJ, UK.

T:(+44) (0)1273 641831
E: J.N.Cole@brighton.ac.uk

Supplementary Methods : Calculating the calorie value of the human body

For this study, data from four adult male human specimens were assimilated to obtain an overall average calorie value. The specific details pertaining to each individual are given below:

Individual 1:

White male, 35 years of age, 70.55 kilograms in weight, 183cm in height. Death was due to a heart attack. Post-mortem revealed passive congestion of both lungs, especially in the lower lobes, and a moderately enlarged heart showing evidence of chronic mitral valvulitis with mitral insufficiency or incompetency. Data from[1].

Individual 2:

White male, 60 years of age, 73.50 kilograms in weight, 172 cm in height. Death was presumably due to a heart attack. Post-mortem revealed arteriosclerosis of the abdominal aorta and coronary arteries, no definite endocarditis, but haemorrhages in edges of mitral and tricuspid valves. Slight enlargement of the heart and moderate fatty degeneration of cardiac musculature and liver along with passive congestion of the lungs. Data from[2].

Individual 3:

Black male, 48 years of age, 62.00 kilograms in weight, 169 cm in height. Death was in a hospital with a diagnosis of heart and vascular disease with subacute bacterial endocarditis. Data from[2].

Individual 4:

White male, 46 years of age, 53.8 kilograms in weight, 168.50 cm in height. Death was due to a skull fracture as a result of a fall. Post-mortem revealed hypostatic congestion of the lungs no other abnormalities were noted. Data from[3].

All data sources[1-3] shared the same original data format of displaying the body composition data as percentages (body weight, fat and protein content of each whole body component, Tables S1-S4). In order to obtain the calorie values, the percentage values had to be converted back to real weights (kilograms – kg, and grams – g). The calorie conversion was attained through the ratio conversion 4:4:9 where 1 gram of protein or carbohydrate equals 4 calories and 1 gram of fat equals 9 calories[4]. The causes of death for each individual were not thought to have to affected the calorific values for each individual. Details pertaining to the conversion of the percentage weight values to real weight values are given below.

Step 1:

The body part weights for each individual were given as a percentage of the whole body (Tables S1-S4). The total body weight of each Individual was known in each case. Therefore, to convert from percentage values back into the raw data weight values the following formula was used:

$$\mathbf{BW/100} \times \mathbf{p} = \mathbf{K}$$

Where:

BW – known body weight

p – percentage body part weight

K – actual body part weight (in kilograms)

Step 2:

Percentage values for fat and protein weights were given in all four instances (Tables S1-S4). Having worked out the weights for each body part, the weight values for protein and fat content were calculated for each body part of each individual using the following formula:

**Fat**	**K/100 x FP = FK**
**Protein**	**K/100 x PP = PK**

Where:

K – actual body part weight (in kilograms)

FP – fat percentage of body part

PP – protein percentage of body part

FK – fat weight of body part (in kilograms)

PK – protein weight of body part (in kilograms)

Step 3:

Fat and protein body part weights for each individual were then converted from kilograms into grams by multiplying the **FK** and **PK** values by 1000 (Tables S1-S4).

Step 4:

The fat and protein gram values for each body part of each individual were multiplied by the respective calorie conversion to obtain calorie values (Tables S1-S4) using the following equation:

**Fat**	**Fg x 9 = FC**
**Protein**	**Pg x 4 = PC**

Where:

Fg – fat weight of body part (in grams)

Pg – protein weight of body part (in grams)

FC – fat calorie value

PC – protein calorie value

The overall results are shown in Tables S1-S4, however, these individual values are difficult to examine in regards to building an average calorie total per body component across the sample base. Therefore, Table S5 shows a comparison of each body component, the calorific value from each of the four individuals and the resulting overall average calorie values. As can be seen from Table S5, the values obtained for the striated/skeletal muscle were for the whole body. To make this nutritional template applicable for the archaeological analysis of hominin remains, specific body parts (upper arm, forearm, thigh, calf, torso and head) would be useful.

The nature of the data obtained from[1-3] could not supply this kind of specific data, thus, in order to obtain these values, the skeletal muscle mass of the respective body parts needed to be estimated. Average weight values of the skeletal muscle for the forearm (consisting of elbow, mid-forearm and wrist), thigh (consisting of symphysis caudal edge, mid-thigh, lower ¼ thigh and knee joint space) and calf (consisting of the knee joint space, upper ⅔ calf, lower ⅓ calf and ankle) were obtained from[5: 1165 – 1166] (Table S6). It is assumed that these results represent both the left and right sides culminated into one value. The data for the weight of the upper arm could not be found as a separate value and therefore had to be calculated by taking the average total weight of the skeletal muscle for the whole arm (7.01 kg[6]) and subtracting the Forearm value from[5] (1.280 kg) from the arm total to get an estimated Upper arm value of 5.73kg. Table S6 shows the results of the nutritional values for the specific limb parts pertaining to skeletal muscle. The workings of those results are explained below:

Step 1:

The average total skeletal muscle mass was obtained from Table S5. The average skeletal muscle weights for the calf, thigh, forearm, upper arm, torso and head were obtained as above with the torso and head values representing the difference between the total skeletal muscle mass minus the total limb value.

Step 2:

The percentages of skeletal muscle mass values were calculated using the following formula:

$$w / sw \times 100 = sm$$

Where:

w – average weight of skeletal muscle limb component (calf, thigh, forearm, upper arm, torso and head)

sw – average weight total skeletal muscle

sm – percentage of total skeletal muscle

Step 3:

The average fat and protein percentage values for the total average skeletal muscle were obtained from Table S5.

Step 4:

The fat and protein gram values for each limb component were multiplied by the respective calorie conversion to obtain calorie values using the following equation:

Fat $FgL \times 9 = FCL$

Protein $PgL \times 4 = PCL$

Where:

FgL – fat weight of limb component (in grams)

PgL – protein weight of limb component (in grams)

FCL – fat calorie value of limb component

PCL – protein calorie value of limb component

It is important to note that the values for the upper arm, forearm, thigh and calf (Table S6) relate to both sides of the body.

In order to construct the nutritional template, the total average calorie values (fat and protein) for each body part (Table S5) and the total average calorie values (fat and protein) for each limb component (Table S6) were combined to produce the nutritional template values shown in Figure S1 and Table 1 representing the nutritional value of an average adult human male.

Supplementary Tables:

Table S1: Data after[1: Table 1] ⫯ - enlarged, ᛁ - congested, ŧ - assumed

**Individual 1: body components**	**% of total body weight**	**Weight (kg)**	**Fat %**	**Protein %**	**Fat (kg)**	**Protein (kg)**	**Fat (g)**	**Protein (g)**	**Calorie (Fat)**	**Calorie (Protein)**	**Total Calorie Value (Fat + Protein)**
Skin	7.81	5.510	13.00	22.19	0.716	1.223	716	1223	6444	4892	11336
Skeleton	14.84	10.470	17.18	18.93	1.799	1.982	1799	1982	16191	7928	24119
Teeth	0.06	0.042		23.00ŧ		0.009		9		36	36
Striated muscle	31.56	22.266	3.35	16.50	0.746	3.674	746	3674	6714	14696	21410
Brain, spinal cord, and nerve trunks	2.52	1.778	12.68	12.06	0.225	0.214	225	214	2025	856	2881
Liver	3.41	2.406	10.35	16.19	0.249	0.389	249	389	2241	1556	3797
Heart⫯	0.69	0.487	9.26	15.88	0.045	0.077	45	77	405	308	713
Lungsᛁ	4.15	2.928	1.54	13.38	0.045	0.392	45	392	405	1568	1973
Spleen	0.19	0.134	1.19	17.81	0.002	0.024	2	24	18	96	114
Kidneys	0.51	0.360	4.01	14.69	0.014	0.053	14	53	126	212	338
Pancreas	0.16	0.113	13.08	12.69	0.015	0.014	15	14	135	56	191
Alimentary tract	2.07	1.460	6.24	13.19	0.091	0.193	91	193	819	772	1591
Adipose tissue	13.63	9.616	42.44	7.06	4.081	0.679	4081	679	36729	2716	39445
Remaining tissues -											
Liquid	3.79	2.674	0.17	5.68	0.005	0.152	5	152	45	608	653
Solid	13.63	9.616	12.39	16.06	1.191	1.544	1191	1544	10719	6176	16895
Contents of alimentary tract	0.80	0.564									
Bile	0.15	0.106									
Hair	0.03	0.021									
Total body, weighing 70.55 kg	100	70.55	Fat total = 12.51% of whole body	Protein total = 14.39% of whole body	9.224	10.619	9224	10619	83016	42476	**125492**

Table S2: Data after[2: Table 1]

Individual 2: body components	% total body weight	Weight (kg)	Fat %	Protein %	Fat (kg)	Protein (kg)	Fat (g)	Protein (g)	Calorie (Fat)	Calorie (Protein)	Total Calorie Value (Fat + Protein)
Skin	6.58	4.836	19.34	27.30	0.935	1.320	935	1320	8415	5280	13695
Skeleton	14.95	10.988	22.04	19.70	2.422	2.165	2422	2165	21798	8660	30458
Teeth											
Striated Muscle	40.22	29.562	9.40	20.81	2.779	6.152	2779	6152	25011	24608	49619
Nerve Tissue	2.13	1.566	9.62	10.76	0.151	0.168	151	168	1359	672	2031
Liver	2.38	1.749	11.48	16.39	0.201	0.287	201	287	1809	1148	2957
Heart	0.60	0.441	11.10	17.5	0.049	0.077	49	77	441	308	749
Lungs	2.21	1.624	1.18	19.24	0.019	0.313	19	313	171	1252	1423
Spleen	0.10	0.074									
Kidney	0.43	0.316	5.80	19.13	0.018	0.060	18	60	162	240	402
Pancreas	0.1	0.074									
Alimentary Tract	1.51	1.110	6.89	10.94	0.076	0.121	76	121	684	484	1168
Adipose Tissue	21.67	15.927	78.35	6.75	12.479	1.075	12479	1075	112311	4300	116611
Remaining Tissue:						0.000					
Liquid	0.5	0.368	0.70	24.64	0.003	0.091	3	91	27	364	391
Solid	5.97	4.388	28.34	15.58	1.244	0.684	1244	684	11187	2736	13923
Bile, bladder, intestinal contents	0.55	0.404									
Hair and nails	0.07	0.051									
Thyroid	0.02	0.015									
Suprarenals	0.01	0.007									
Total body weighing 73.5 kg	100	73.5	Fat total = 27.93% of whole body	Protein total = 17.17% of whole body	20.376	12.513	20376	12513	183375	50052	**233427**

Table 3: Data after[2: Table 1]

Individual 3: body components	% total body weight	Weight (kg)	Fat %	Protein %	Fat (kg)	Protein (kg)	Fat (g)	Protein (g)	Calorie (Fat)	Calorie (Protein)	Total Calorie Value (Fat + Protein)
Skin	9.49	5.884	4.83	23.10	0.284	1.359	284	1359	2556	5436	7992
Skeleton	16.68	10.342	10.07	20.82	1.041	2.153	1041	2153	9369	8612	17981
Teeth	0.07	0.043									
Striated Muscle	42.53	26.369	2.22	20.60	0.585	5.432	585	5432	5265	21728	26993
Nerve Tissue	2.41	1.494	9.30	12.07	0.139	0.180	139	180	1251	720	1971
Liver	3.39	2.102	2.50	18.74	0.053	0.394	53	394	477	1576	2053
Heart	0.86	0.533	2.72	18.56	0.015	0.099	15	99	135	396	531
Lungs	3.07	1.903	1.02	16.41	0.019	0.312	19	312	171	1248	1419
Spleen	0.42	0.260	0.85	19.25	0.002	0.050	2	50	18	200	218
Kidney	0.70	0.434	1.76	17.24	0.008	0.075	8	75	72	300	372
Pancreas	0.20	0.124									
Alimentary Tract	2.15	1.333	1.34	14.89	0.018	0.198	18	198	162	792	954
Adipose Tissue	5.18	3.212	4.24	12.76	0.136	0.410	136	410	1224	1640	2864
Remaining Tissue:											
Liquid	1.23	0.763	0.30	18.74	0.002	0.143	2	143	18	572	590
Solid	10.45	6.479	5.22	19.32	0.338	1.252	338	1252	3042	5008	8050
Bile, bladder, intestinal contents	1.05	0.651									
Hair and nails	0.03	0.019									
Thyroid	0.03	0.019									
Suprarenals	0.06	0.037									
Total body weighing 62 kg	100.00	62.0	Fat total = 4.32% of whole body	Protein total = 19.73% of whole body	2.641	12.058	2640	12057	23760	48228	**71988**

Table S4: Data after[3: Table 1], † - Chemical Composition Assumed, ‡ - Congested with Blood

**Individual 4: body components**	**% total body weight**	**Weight (kg)**	**Fat %**	**Protein %**	**Fat (kg)**	**Protein (kg)**	**Fat (g)**	**Protein (g)**	**Calorie (Fat)**	**Calorie (Protein)**	**Total Calorie Value (Fat + Protein)**
Skin	6.33	3.406	14.23	27.33	0.485	0.931	485	931	4365	3724	8089
Skeleton	17.58	9.458	25.04	19.71	2.368	1.864	2368	1864	21312	7456	28768
Teeth	0.08	0.043		23.00†		0.009		9		36	36
Striated Muscle	39.76	21.391	6.60	21.94	1.412	4.693	1412	4693	12708	18772	31480
Brian, spinal cord, nerve trunks	2.99	1.609	12.35	11.50	0.199	0.185	199	185	1791	740	2531
Liver	2.34	1.259	3.11	22.24	0.039	0.280	39	280	351	1120	1471
Heart	0.52	0.280	16.58	17.48	0.046	0.049	46	49	414	196	610
Lungs†	3.30	1.775	1.32	19.20	0.023	0.341	23	341	207	1364	1571
Spleen‡	0.11	0.059	1.19	17.81	0.001	0.011	1	11	9	44	53
Kidneys	0.51	0.274	7.18	19.28	0.020	0.053	20	53	180	212	392
Pancreas‡	0.14	0.075	13.08	12.69	0.010	0.010	10	10	90	40	130
Alimentary Tract	1.86	1.001	9.17	12.77	0.092	0.128	92	128	828	512	1340
Adipose Tissue	11.37	6.117	71.57	5.85	4.378	0.358	4378	358	39402	1432	40834
Remaining Tissue:											
Liquid	0.59	0.317	2.55	13.58	0.008	0.043	8	43	72	172	244
Solid	11.43	6.149	22.47	17.28	1.382	1.063	1382	1064	12438	4256	16694
Bile, content of bladder and alimentary tract	0.99	0.533									
Hair and nails	0.1	0.054									
Total body weighing 53.8 kg	100	53.8	Fat total = 19.44% of whole body	Protein total = 18.62% of whole body	10.462	10.016	10463	10019	94167	40076	**134243**

Table S5: Showing the weight to calorie conversions for individual components of the human body. Data from Tables S1-S4.

Individual	Body Component	% total body weight	Weight (kg)	Fat %	Protein %	Fat (g)	Protein (g)	Calorie Value Fat	Calorie Value Protein	Total Calorie Value (Fat + Protein)
1	Skin	7.81	5.51	13.00	22.19	716.00	1223.00	6444.00	4892.00	11336.00
2	Skin	6.58	4.84	19.34	27.30	935.00	1320.00	8415.00	5280.00	13695.00
3	Skin	9.49	5.88	4.83	23.10	284.00	1359.00	2556.00	5436.00	7992.00
4	Skin	6.33	3.41	14.23	27.33	485.00	931.00	4365.00	3724.00	8089.00
Total		*30.21*	*19.64*	*51.40*	*99.92*	*2420.00*	*4833.00*	*21780.00*	*19332.00*	*41112.00*
Average		*7.55*	*4.91*	*12.85*	*24.98*	*605.00*	*1208.25*	*5445.00*	*4833.00*	***10278.00***
1	Skeleton	14.84	10.47	17.18	18.93	1799.00	1982.00	16191.00	7928.00	24119.00
2	Skeleton	14.95	10.99	22.04	19.70	2422.00	2165.00	21798.00	8660.00	30458.00
3	Skeleton	16.68	10.34	10.07	20.82	1041.00	2153.00	9369.00	8612.00	17981.00
4	Skeleton	17.58	9.46	25.04	19.71	2368.00	1864.00	21312.00	7456.00	28768.00
Total		*64.05*	*41.26*	*74.33*	*79.16*	*7630.00*	*8164.00*	*68670.00*	*32656.00*	*101326.00*
Average		*16.01*	*10.31*	*18.58*	*19.79*	*1907.50*	*2041.00*	*17167.50*	*8164.00*	***25331.50***
1	Teeth	0.06	0.04		23.00		9.00		36.00	36.00
4	Teeth	0.08	0.04		23.00		9.00		36.00	36.00
Total		*0.14*	*0.09*		*46.00*		*18.00*		*72.00*	*72.00*
Average		*0.07*	*0.04*		*23.00*		*9.00*		*36.00*	***36.00***
1	Skeletal muscle	31.56	22.27	3.35	16.50	746.00	3674.00	6714.00	14696.00	21410.00
2	Skeletal muscle	40.22	29.56	9.40	20.81	2779.00	6152.00	25011.00	24608.00	49619.00
3	Skeletal muscle	42.53	26.37	2.22	20.60	585.00	5432.00	5265.00	21728.00	26993.00
4	Skeletal muscle	39.76	21.39	6.60	21.94	1412.00	4693.00	12708.00	18772.00	31480.00
Total		*154.07*	*99.59*	*21.57*	*79.85*	*5522.00*	*19951.00*	*49698.00*	*79804.00*	*129502.00*
Average		*38.52*	*24.90*	*5.39*	*19.96*	*1380.50*	*4987.75*	*12424.50*	*19951.00*	***32375.50***

1	Brain, spinal cord, nerve trunks	2.52	1.78	12.68	12.06	225.00	214.00	2025.00	856.00	2881.00
4	Brain, spinal cord, nerve trunks	2.99	1.61	12.35	11.50	199.00	185.00	1791.00	740.00	2531.00
Total		*5.51*	*3.39*	*25.03*	*23.56*	*424.00*	*399.00*	*3816.00*	*1596.00*	*5412.00*
Average		*2.76*	*1.69*	*12.52*	*11.78*	*212.00*	*199.50*	*1908.00*	*798.00*	***2706.00***
2	Nerve Tissue	2.13	1.57	9.62	10.76	151.00	168.00	1359.00	672.00	2031.00
3	Nerve Tissue	2.41	1.49	9.30	12.07	139.00	180.00	1251.00	720.00	1971.00
Total		*4.54*	*3.06*	*18.92*	*22.83*	*290.00*	*348.00*	*2610.00*	*1392.00*	*4002.00*
Average		*2.27*	*1.53*	*9.46*	*11.42*	*145.00*	*174.00*	*1305.00*	*696.00*	***2001.00***
1	Liver	3.41	2.41	10.35	16.19	249.00	389.00	2241.00	1556.00	3797.00
2	Liver	2.38	1.75	11.48	16.39	201.00	287.00	1809.00	1148.00	2957.00
3	Liver	3.39	2.10	2.50	18.74	53.00	394.00	477.00	1576.00	2053.00
4	Liver	2.34	1.26	3.11	22.24	39.00	280.00	351.00	1120.00	1471.00
Total		*11.52*	*7.52*	*27.44*	*73.56*	*542.00*	*1350.00*	*4878.00*	*5400.00*	*10278.00*
Average		*2.88*	*1.88*	*6.86*	*18.39*	*135.50*	*337.50*	*1219.50*	*1350.00*	***2569.50***
1	Heart	0.69	0.49	9.26	15.88	45.00	77.00	405.00	308.00	713.00
2	Heart	0.60	0.44	11.10	17.50	49.00	77.00	441.00	308.00	749.00
3	Heart	0.86	0.53	2.72	18.56	15.00	99.00	135.00	396.00	531.00
4	Heart	0.52	0.28	16.58	17.48	46.00	49.00	414.00	196.00	610.00
Total		*2.67*	*1.74*	*39.66*	*69.42*	*155.00*	*302.00*	*1395.00*	*1208.00*	*2603.00*
Average		*0.67*	*0.44*	*9.92*	*17.36*	*38.75*	*75.50*	*348.75*	*302.00*	***650.75***
1	Lungs	4.15	2.93	1.54	13.38	45.00	392.00	405.00	1568.00	1973.00
2	Lungs	2.21	1.62	1.18	19.24	19.00	313.00	171.00	1252.00	1423.00
3	Lungs	3.07	1.90	1.02	16.41	19.00	312.00	171.00	1248.00	1419.00
4	Lungs	3.30	1.78	1.32	19.20	23.00	341.00	207.00	1364.00	1571.00

Total		*12.73*	*8.23*	*5.06*	*68.23*	*106.00*	*1358.00*	*954.00*	*5432.00*	*6386.00*
Average		*3.18*	*2.06*	*1.27*	*17.06*	*26.50*	*339.50*	*238.50*	*1358.00*	***1596.50***
1	Spleen	0.19	0.13	1.19	17.81	2.00	24.00	18.00	96.00	114.00
3	Spleen	0.42	0.26	0.85	19.25	2.00	50.00	18.00	200.00	218.00
4	Spleen □	0.11	0.06	1.19	17.81	1.00	11.00	9.00	44.00	53.00
Total		*0.72*	*0.45*	*3.23*	*54.87*	*5.00*	*85.00*	*45.00*	*340.00*	*385.00*
Average		*0.24*	*0.15*	*1.08*	*18.29*	*1.67*	*28.33*	*15.00*	*113.33*	***128.33***
1	Kidneys	0.51	0.36	4.01	14.69	14.00	53.00	126.00	212.00	338.00
2	Kidney	0.43	0.32	5.80	19.13	18.00	60.00	162.00	240.00	402.00
3	Kidney	0.70	0.43	1.76	17.24	8.00	75.00	72.00	300.00	372.00
4	Kidneys	0.51	0.27	7.18	19.28	20.00	53.00	180.00	212.00	392.00
Total		*2.15*	*1.38*	*18.75*	*70.34*	*60.00*	*241.00*	*540.00*	*964.00*	*1504.00*
Average		*0.54*	*0.35*	*4.69*	*17.59*	*15.00*	*60.25*	*135.00*	*241.00*	***376.00***
1	Pancreas	0.16	0.11	13.08	12.69	15.00	14.00	135.00	56.00	191.00
4	Pancreas□	0.14	0.08	13.08	12.69	10.00	10.00	90.00	40.00	130.00
Total		*0.30*	*0.19*	*26.16*	*25.38*	*25.00*	*24.00*	*225.00*	*96.00*	*321.00*
Average		*0.15*	*0.09*	*13.08*	*12.69*	*12.50*	*12.00*	*112.50*	*48.00*	***160.50***
1	Alimentary tract	2.07	1.46	6.24	13.19	91.00	193.00	819.00	772.00	1591.00
2	Alimentary Tract	1.51	1.11	6.89	10.94	76.00	121.00	684.00	484.00	1168.00
3	Alimentary Tract	2.15	1.33	1.34	14.89	18.00	198.00	162.00	792.00	954.00
4	Alimentary Tract	1.86	1.00	9.17	12.77	92.00	128.00	828.00	512.00	1340.00
Total		*7.59*	*4.90*	*23.64*	*51.79*	*277.00*	*640.00*	*2493.00*	*2560.00*	*5053.00*
Average		*1.90*	*1.23*	*5.91*	*12.95*	*69.25*	*160.00*	*623.25*	*640.00*	***1263.25***
1	Adipose tissue	13.63	9.62	42.44	7.06	4081.00	679.00	36729.00	2716.00	39445.00
2	Adipose Tissue	21.67	15.93	78.35	6.75	12479.00	1075.00	112311.00	4300.00	116611.00
3	Adipose Tissue	5.18	3.21	4.24	12.76	136.00	410.00	1224.00	1640.00	2864.00
4	Adipose Tissue	11.37	6.12	71.57	5.85	4378.00	358.00	39402.00	1432.00	40834.00

Total		*51.85*	*34.87*	*196.60*	*32.42*	*21074.00*	*2522.00*	*189666.00*	*10088.00*	*199754.00*
Average		*12.96*	*8.72*	*49.15*	*8.11*	*5268.50*	*630.50*	*47416.50*	*2522.00*	***49938.50***
	Remaining Tissue									
1	Liquid	3.79	2.67	0.17	5.68	5.00	152.00	45.00	608.00	653.00
2	Liquid	0.50	0.37	0.70	24.64	3.00	91.00	27.00	364.00	391.00
3	Liquid	1.23	0.76	0.30	18.74	2.00	143.00	18.00	572.00	590.00
4	Liquid	0.59	0.32	2.55	13.58	8.00	43.00	72.00	172.00	244.00
Total		*6.11*	*4.12*	*3.72*	*62.64*	*18.00*	*429.00*	*162.00*	*1716.00*	*1878.00*
Average		*1.53*	*1.03*	*0.93*	*15.66*	*4.50*	*107.25*	*40.50*	*429.00*	***469.50***
	Remaining Tissue									
1	Solid	13.63	9.62	12.39	16.06	1191.00	1544.00	10719.00	6176.00	16895.00
2	Solid	5.97	4.39	28.34	15.58	1244.00	684.00	11187.00	2736.00	13923.00
3	Solid	10.45	6.48	5.22	19.32	338.00	1252.00	3042.00	5008.00	8050.00
4	Solid	11.43	6.15	22.47	17.28	1382.00	1064.00	12438.00	4256.00	16694.00
Total		*41.48*	*26.63*	*68.42*	*68.24*	*4155.00*	*4544.00*	*37386.00*	*18176.00*	*55562.00*
Average		*10.37*	*6.66*	*17.11*	*17.06*	*1038.75*	*1136.00*	*9346.50*	*4544.00*	***13890.50***

Table S6: Showing the weight to calorie conversions for skeletal muscle including limbs. * Data from[5: Table 5] using average weight values between CT and New DEXA-SM models (combined left and right).** Data from[6: Table 2] using average arm weight totals (combined left and right across the whole sample) = 7.01 kg, Forearm value from[5] (1.280 kg) subtracted from arm total (7.01 kg) to get an estimated Upper arm value of 5.73kg.

**Body Component**	**Weight (kg)**	**Skeletal Muscle %**	**Fat (g)**	**Protein (g)**	**Calorie Value Fat**	**Calorie Value Protein**	**Calorie Value Total (Fat + Protein)**
Average total skeletal muscle	24.897	100	1380.500	4987.750	12424.500	19951.000	32375.500
Average torso and head skeletal muscle	4.167	16.737	231.054	834.798	2079.483	3339.190	5418.673
Average total of limb muscles	20.730	83.263	1149.446	4152.952	10345.017	16611.810	26956.827
Calf (both)	*3.450	13.857	191.297	691.157	1721.674	2764.628	4486.303
Thigh (both)	*10.270	41.250	569.456	2057.444	5125.100	8229.777	13354.877
Forearm (both)	*1.280	5.141	70.974	256.429	638.766	1025.717	1664.483
Upperarm (both)	**5.730	23.015	317.720	1147.922	2859.476	4591.687	7451.163

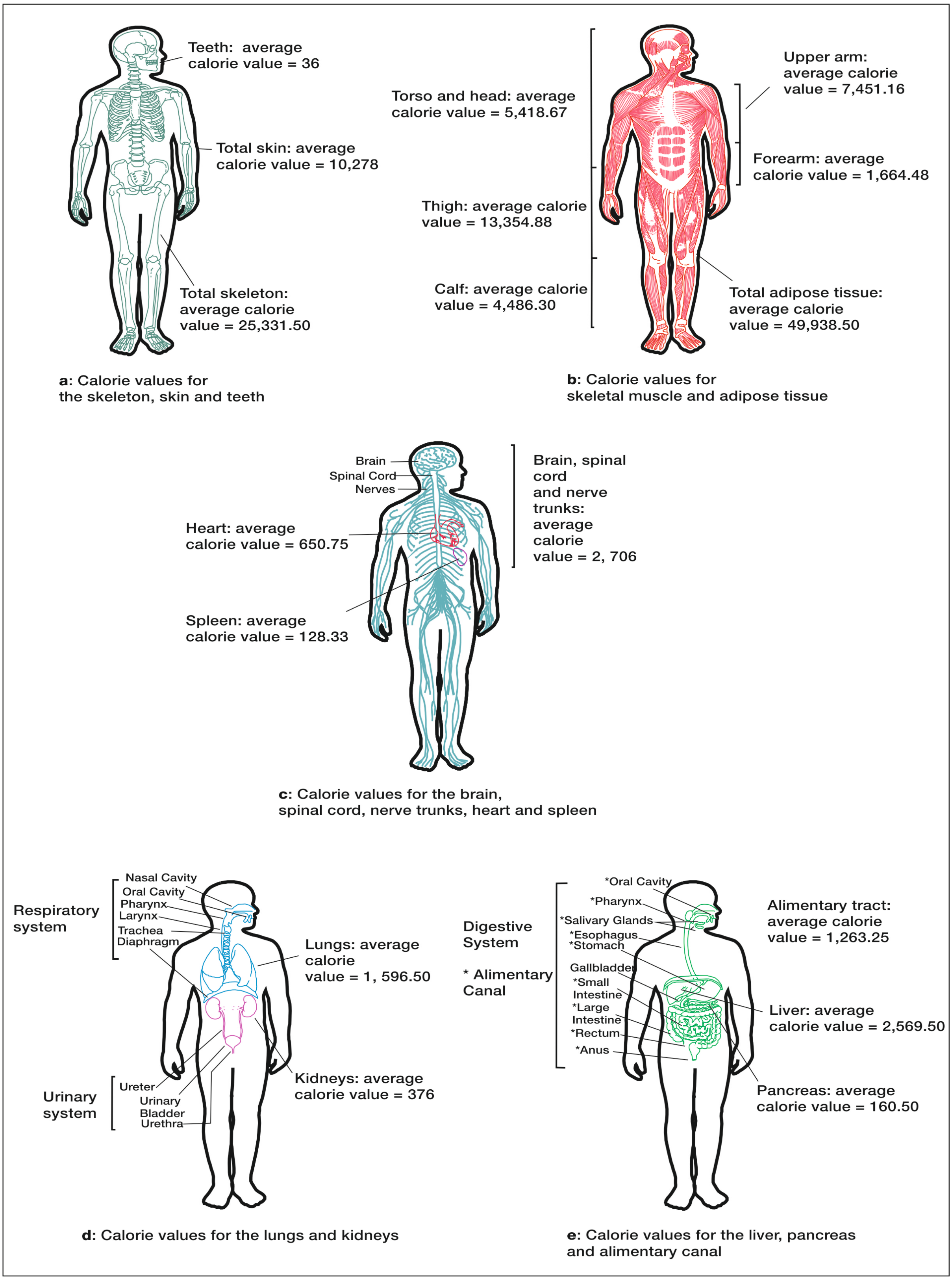

Figure S1: Showing the calorific value for different components of the human body. Image subdivided into sections for ease of reference: a = the skeleton, skin and teeth; b = skeletal muscle and adipose tissue; c = brain, spinal cord, nerve trunks, heart and spleen; d = lungs and kidneys; e = liver, pancreas and alimentary canal. Figure represents a simplified summary from Table 1 and Tables S1-S6.

Supplementary References Cited:

1 Mitchell, H. H., Hamilton, T. S., Steggerda, F. R. & Bean, H. W. The Chemical Composition of the Adult Human Body and its bearing on the Biochemistry of Growth. *Journal of Biological Chemistry* **158**, 625-637 (1945).

2 Forbes, R. M., Mitchell, H. H. & Cooper, R. H. Further Studies on the Gross Composition and Mineral Elements of the Adult Human Body. *Journal of Biological Chemistry* **223**, 969-975 (1956).

3 Forbes, R. M., Cooper, R. H. & Mitchell, H. H. The Composition of the Adult Human Body as Determined by Chemical Analysis. *Journal of Biological Chemistry* **203**, 359-366 (1953).

4 USDA. in *United States Department of Agriculture National Nutrient Database for Standard Reference, Release 18* (2005).

5 Wang, W. *et al.* Regional skeletal muscle measurement: evaluation of new dual energy X-ray absorptiometry model. *Journal of Applied Physiology* **87**, 1163-1171 (1999).

6 Sugawara, J. *et al.* Age-related reductions in appendicular skeletal muscle mass: association with habitual aerobic exercise status. *Clinical Physiology and Functional Imaging* **22**, 169-172 (2002).
